# A Metabolite-Wide Association Study Of Frailty In Over 200,000 Adults

**DOI:** 10.1093/geroni/igaf122.2362

**Published:** 2025-12-31

**Authors:** Julian Mutz

**Affiliations:** King’s College London, London, England, United Kingdom

## Abstract

Frailty is an age-related medical syndrome associated with increased mortality risk. Identifying metabolomic profiles linked to frailty may provide insights into its biological causes and consequences. The UK Biobank is a multi-centre observational health study that recruited over half a million middle-aged and older adults. The Nightingale Health platform was used to quantify circulating plasma metabolites. We derived the two most common measures of frailty: the frailty phenotype and the frailty index. The analytical sample included over 200,000 participants (mean age = 56.44 years, SD = 8.12). Most metabolite levels varied with chronological age, showing considerable evidence of non-linear relationships. Of the metabolites statistically significantly associated with age (P < 0.05/168), 95% were also associated with the frailty index and 89% with the frailty phenotype. A metabolite-predicted age (MileAge) older than one’s chronological age was associated with higher frailty index scores (β = 0.023, 95% CI 0.021–0.024, P < 0.001, comparing individuals with a MileAge delta at least one standard deviation above and below the mean). This group difference was comparable to an 18.3-year chronological age difference in frailty index scores. Individuals with a metabolite-predicted age older than their chronological age were also more likely to be physically frail (OR = 1.29, 95% CI 1.23–1.35, P < 0.001). These findings highlight a considerable overlap in the metabolites associated with age and frailty. Metabolite-based age predictions are correlated with both the frailty index and the frailty phenotype, suggesting they have potential for identifying individuals at risk of developing frailty.

